# Neuroplasticity Underlying the Comorbidity of Pain and Depression

**DOI:** 10.1155/2015/504691

**Published:** 2015-02-25

**Authors:** Lisa Doan, Toby Manders, Jing Wang

**Affiliations:** ^1^Department of Anesthesiology, New York University School of Medicine, New York, NY 10016, USA; ^2^Department of Neuroscience and Physiology, New York University School of Medicine, New York, NY 10016, USA

## Abstract

Acute pain induces depressed mood, and chronic pain is known to cause depression. Depression, meanwhile, can also adversely affect pain behaviors ranging from symptomology to treatment response. Pain and depression independently induce long-term plasticity in the central nervous system (CNS). Comorbid conditions, however, have distinct patterns of neural activation. We performed a review of the changes in neural circuitry and molecular signaling pathways that may underlie this complex relationship between pain and depression. We also discussed some of the current and future therapies that are based on this understanding of the CNS plasticity that occurs with pain and depression.

## 1. Introduction

Pain encompasses sensory, cognitive, and most importantly affective components. The affective component of pain includes feelings of annoyance, sadness, anxiety, and depression in response to a noxious stimulus. In particular, depression and pain share a high degree of comorbidity, and a large number of studies have examined the close relationship between pain and depression.

Acute pain can adversely affect mood following surgery. In the immediate postoperative period, the rate of depression has been reported to be between 21 and 50% in study populations with low (0–11.8%) levels of preoperative depression. Indeed, postoperative pain intensity is correlated with the degree of depressive symptoms [1–3]. High postoperative depression scores have also been associated with increased length of stay and poor functional outcomes after surgeries [3, 4]. Preoperative psychological factors may also negatively affect the resolution of acute pain. Preoperative anxiety and catastrophization are two well-studied risk factors for the development of chronic postsurgical pain [5]. Both of these factors are known to lead to worsening depressed mood in the postoperative period.

Chronic pain and comorbid depression are frequently encountered clinically. In patients treated for depression, the prevalence of chronic pain is reported to be 51.8–59.1% [6–8]. Longitudinal studies have shown that depression is a risk factor for the onset of disabling or chronic pain [9, 10]. Conversely, in patients with chronic pain, the mean prevalence of major depression is reported to be between 18 and 85%, depending on the practice setting [11, 12]. In fact, pain is a major risk factor for the development of depression. In a longitudinal, cohort study with 12-year follow-up, pain at baseline as well as the severity and chronicity of pain was statistically significantly associated with the onset of depression [13].

Pain adversely affects the prognosis and treatment of depression and vice versa. There is a significant correlation between the severity of pain and the degree of depression [14]. In a study examining the long-term course of depression, greater severity of pain at baseline, greater number of pain locations, and longer duration of pain all significantly increased the risk of still having depression after two years [15]. Baseline pain severity prior to the initiation of antidepressant treatment has also been shown to be a strong negative predictor of treatment response [16]. At the same time, depression also adversely affects prognosis in the treatment of chronic pain. Patients with chronic pain and depression report more pain complaints and increased severity and longer duration of pain symptoms [16]. Some studies have reported that patients with comorbid pain and depression have poorer response to pain treatment than nondepressed patients [17]. Comorbid pain and depression also lead to significant functional impairments. In a cross-sectional study, patients with major depressive disorder with chronic pain are found to be 2.1–4.6 times more likely to report interference in activities of daily living and family and social interactions than depressed patients without pain. They have also been found to be more likely to take sick leave because of pain [18].

Thus, a wealth of clinical data suggests a high degree of comorbidity between pain and depression. Basic and translational studies utilizing imaging as well as animal models have begun to unravel the mechanistic basis for the relationship between pain and depression. In this review, we will examine the current understanding of the circuit and molecular plasticity that underlie this complex relationship and how such understanding can lead to successful therapies.

## 2. Neuroimaging Evidence for the Plasticity of Pain and Depression Networks

### 2.1. Identification of Pain and Depression Circuits Based on Human Imaging Studies

**Figure 1:**
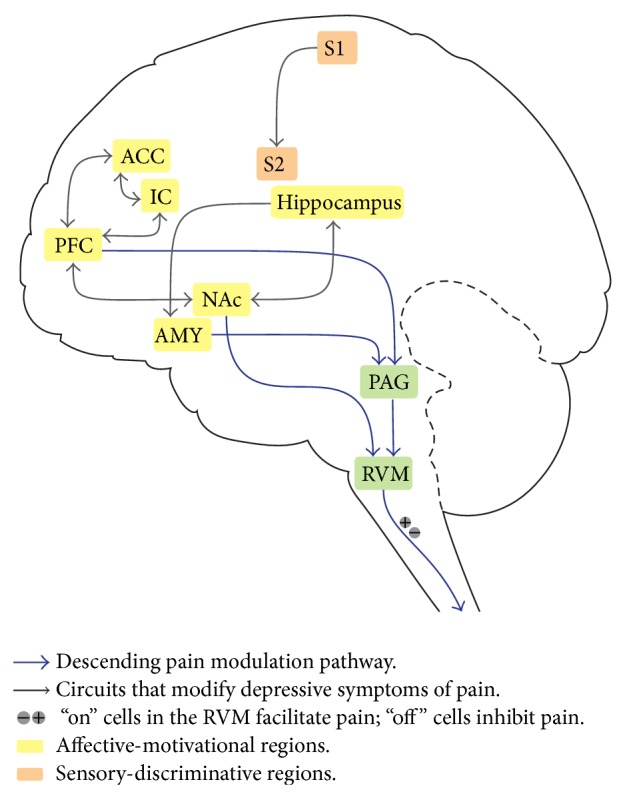
Brain regions and circuits implicated in the comorbidity between pain and depression. ACC: anterior cingulate cortex; AMY: amygdala; IC: insular cortex; NAc: nucleus accumbens; PAG: periaqueductal gray; PFC: prefrontal cortex; RVM: rostral ventromedial medulla; S1: primary somatosensory cortex; S2: secondary somatosensory cortex.

In imaging studies of acute experimental pain in human subjects, areas most commonly activated include the primary somatosensory (S1) and secondary somatosensory cortex (S2), anterior cingulate cortex (ACC), insular cortex (IC), prefrontal cortex (PFC), thalamus, nucleus accumbens (NAc), and amygdala [19–21]. S1 and S2 activations contribute to the sensory-discriminative dimension of pain. The ACC, PFC, IC, NAc, and amygdala, meanwhile, have been implicated in the affective component of pain (Figure 1). Distinct alterations in brain structure and activity, meanwhile, occur with chronic pain. For example, reductions in gray matter volume are observed in the IC, ACC, and PFC, areas involved in the emotional and cognitive aspects of pain [22].

Several studies have also directly examined brain changes in the transition from acute to chronic pain. Brain activity in patients with back pain for approximately two months showed activations in the IC, thalamus, ACC, and PFC [23]. In patients with back pain for more than ten years, meanwhile, abnormal activations in the perigenual ACC, medial PFC (mPFC), and amygdala are observed in response to pain. Thus, the transition from acute to chronic pain may be accompanied by a shift from the sensory to affective-emotional circuitry for pain [24]. Additionally, functional connectivity of the NAc with the PFC has been found to be higher in patients with persistent back pain, indicating that the reward circuitry may also play a role in this switch from sensory to affective focus in chronic pain [23, 24].

Interestingly, the regions and circuits identified in acute and particularly chronic pain studies closely mirror those found in studies of depression. Thus, areas most commonly found to be dysregulated in depressed patients include the PFC, ACC, NAc, hippocampus, and amygdala [25, 26]. Gray matter volume loss and alterations in activity have been found in these areas, similar to changes that occur with chronic pain. The PFC and ACC are both involved in the processing of negative mood and affect and thus are implicated in depression studies. The NAc, in addition, may be involved in symptoms such as anhedonia. The hippocampus and amygdala, meanwhile, play roles in the formation and retrieval of negative emotional memory that is associated with pain and depression.

More recently, fMRI studies of resting state networks, during task-free settings, have been done to examine changes in patients with chronic pain and depression. Compared to healthy controls, the default mode network (DMN) is altered in patients with chronic pain, with greater representation in the precuneus and posterior cingulate cortex and less representation in the PFC [27]. Correlation of the PFC with the IC is increased, and connectivity of the PFC with posterior portions of the DMN is decreased in patients with chronic pain. The DMN consists of a set of brain regions active at rest and deactivated during a task. The precise function of the DMN is unclear, but it may be involved in the regulation of attention and arousal. Altered connectivity of the mPFC and IC may reflect changes in emotional circuits associated with chronic pain [27]. Similar changes in the DMN are also seen in depressed patients. For example, in a resting state fMRI study of depressed patients, patterns of functional connectivity in the DMN and affective network based on multivariate pattern analysis are distinct between depressed patients and healthy controls. Thus, connections involving the PFC and ACC are most likely to be altered in the comorbidity between chronic pain and depression [28].

### 2.2. Brain Imaging of Physical and Emotional Pain

It is thought that the concept of suffering is central to the experience of pain as well as the experience of depression [29–31]. Hence, depression has been interpreted as a state of being emotional as opposed to physical pain. A number of imaging studies have addressed this possibility. In healthy subjects, the pattern of brain activity observed with emotional pain, such as intense social rejection which is known to cause depressed mood, is similar to activations in response to exposure to experimental pain, including somatosensory as well as affective areas [32, 33]. A recent study, however, suggests that although there is commonality at the gross anatomical level, distinct networks may actually underlie acute physical versus emotional pain [34]. This study finds that experimental pain and intense social rejection activated similar brain regions in human subjects, including S2, IC, thalamus, and periaqueductal gray (PAG). However, multivariate pattern analysis showed distinct physical and emotional representations in these regions. Nevertheless, some shared representations were found in areas not primarily involved with sensory pain processing, including the left parahippocampal and fusiform gyri, retrosplenial cortex, posterior cingulate cortex, and striatum. These shared representations may thus be associated with behavioral context, memory, and motivation, and they may form the network link between depression and pain [35].

There is evidence that depressed mood or other negative emotional states can also modulate sensory neural responses. In an fMRI study, subjects were exposed to nonpainful visceral stimulation during neutral and negative emotional contexts. Significantly greater activations were noted in the ACC and anterior IC during increasingly greater negative emotional contexts and greater levels of reported anxiety that is commonly found in depressed patients [36].

### 2.3. The Complexity of the Chronic Pain and Depression Networks

The interaction between chronic pain and depression is likely more complex than assessing acute, experimental conditions. As noted above, in the transition from acute to chronic back pain, a shift from nociceptive to emotional circuitry has been observed [24]. Similar brain regions may be dysregulated in both chronic pain and depression, including areas involved in emotional and reward processing such as the ACC, IC, PFC, and NAc. The DMN is altered in chronic pain and depression. However, in their study of patients with chronic pain, Baliki et al. investigated the effect of comorbid depression on spatial and connectivity properties of resting state networks and found only low correlations between depression and their network connectivity measurements [27]. Further investigation is thus required to explore the relationship between chronic pain and depression at the circuit level. Depressive symptomatology has a wide spectrum, and different brain areas may regulate different symptom clusters. Similarly, chronic pain varies in etiology and disability, and different conditions may have distinct links with depressive symptomatology.

## 3. Animal Models for the Study of Pain and Depression

Whereas imaging studies have established evidence of plasticity at the anatomic and circuit level that underlie the relationship between pain and depression, they do not reveal mechanistic insights into the cause and effect relationship between changes in neurocircuitry and the pathology of pain and depression. Rodent models, on the other hand, have enabled preclinical investigation into the mechanisms and symptoms of pain-induced depression. Two well-developed behavioral assays for depression in rodents are the sucrose preference test (SPT) and the forced swim test (FST) [37]. The SPT is an assay for anhedonia, a salient feature of clinical depression. The SPT involves measuring animals' preference for sucrose solution compared with water. Rodents naturally prefer sucrose over water. Depressed rodents, however, display a decreased preference for sucrose and hence symptoms of anhedonia. The FST, meanwhile, assesses behavioral despair, another important feature of depression. On this test, a rodent is placed into a water tank for fifteen minutes. It is then returned to the same tank 24 hours later. If the rodent is depressed, it will display a decreased motivation to swim during the second session. Other related tests that can be used to test depression-like behaviors include the open field test, elevated plus maze, and novelty-suppressed feeding test which are assays for anxiety-like behaviors that often accompany depression. Another behavioral assay, the conditioned place avoidance/preference test (CPA/CPP), allows additional insight into the aversive and motivational aspects of animals in pain or depressed states. In the CPA and CPP, an animal with no preexisting preference is trained to associate one chamber in a two-chamber set with either a rewarding or aversive stimulus, such as pain and or pain relief, or a pro- or antidepressant, and to associate the other chamber with the control condition. After a period of associative training, the animal is then allowed to freely choose the chamber it prefers or avoids. This test has been used to test the efficacy of both antidepressants and analgesics [38–41].

Models for depression and pain are both very well established [37, 42–45]. On the one hand, widely accepted models for depression already rely on pain as a stressor to induce depression-like symptoms. In the social defeat model, for example, smaller animals are subjected to physical interactions with larger, more aggressive individuals, and pain resulting from such aggressive physical interactions plays an important role in inducing subsequent depression-like behaviors [37, 46, 47]. In a study combining the social defeat model with a neuropathic pain model in rats, five-day exposure to social stress was found to ameliorate pain sensitivity in chronic pain, but ten-day exposure worsened pain thresholds [46]. Conversely, a study in which mice were housed in either an impoverished or enriched environment after developing chronic neuropathic pain symptoms demonstrated that the enriching environment improved chronic pain symptoms [48].

At the same time, current rodent models for pain, including acute and persistent pain models, have been shown to induce depression-like behaviors. Incision models appear to mimic acute surgical pain in humans [43]. Incisions penetrating the skin, fascia, and muscle, such as the plantar incision model using the rodent hind paw, the gastrocnemius incision model using the posterior surface of the rodent hind limb, and the tail incision model, reliably induce allodynia, a pain behavior in response to a nonnoxious stimulus [5]. Moreover, hyperalgesia, increased sensitivity to a noxious stimulus, can develop around the site of the incision. Acute incisional pain has been suggested to induce temporary depression-like symptoms as manifested by decreased sucrose preference, and these depressive symptoms resolve in a time frame similar to that of the recovery of sensory symptoms [49].

Persistent pain models are capable of inducing longer lasting depressive symptoms. An inflammatory model of pain, in which complete Freund's adjuvant (CFA) is injected into the hind paw in rodents, reliably induces sensory symptoms of chronic inflammatory pain lasting at least two weeks [44]. This CFA model has also been shown to induce depression-like symptoms. After CFA injection, rats with inflammatory pain have been found to show significantly diminished sucrose preference compared to control animals, and this depressive phenotype can last at least two to five weeks [50, 51]. In addition, CFA-treated rats can also develop behavioral despair, another feature of depression [52, 53]. Finally, inflammatory pain has been shown to induce place avoidance in rats [54, 55]. In this test, rats stayed away from the chamber associated with inflammatory pain, suggesting that pain serves as an aversive stimulus that the rats actively avoid. Similarly, acute or repeated stress has been shown as an aversive stimulus in the CPA test in a number of rodent depression studies [37, 56]. Thus, studies using the CPA have demonstrated that pain, similar to psychological stressors known to cause depression, can serve as powerful aversive signals to alter behavior.

Chronic neuropathic pain models mimic human chronic pain-induced depression more closely than acute or inflammatory pain. The most commonly used models of neuropathic pain employ peripheral nerve injury, and these include the chronic constriction injury (CCI), by which ligatures are wrapped around the sciatic nerve, the sciatic nerve ligation model (SNL) of mononeuropathy, by which the L4 spinal nerve is ligated, and the spared nerve injury (SNI) model, by which two of the three terminal branches of the sciatic nerve, the tibial and common peroneal nerves, are axotomized [42, 57]. SNI has been shown to induce anhedonia as well as behavioral despair in rats, as measured by the SPT and FST [49, 58]. Certain types of pain treatment, meanwhile, have been shown to decrease these depressive symptoms [53]. SNL, likewise, has been shown to cause depressive symptoms as well as anxiety in rodents [59, 60].

Thus, animal models for depression have already employed pain as an element of stress to induce depressive symptoms. More importantly, various rodent pain models have been validated to induce depression-like behaviors and hence serve as valuable models for the study of the comorbidity between pain and depression.

## 4. Neurocircuit Plasticity That Underlies the Comorbidity of Pain and Depression

The advancement of modern systems-level neuroscience tools has enabled the identification of brain regions involved in pain and depression processing and the investigation into their structure and function. Pain is a complex, multidimensional experience that involves the potential recruitment of a large, bilateral network of brain regions. These components may become activated dynamically depending on a number of factors: context, stimulus, cognition, and emotion [22, 61]. Recent efforts to identify a so-called pain signature have resulted in the identification of regions which are most essential to experiencing pain. These regions include the thalamus, the cortex, including the ACC, IC, S1, S2, NAc, and the PAG [19, 21]. A much larger system involves the function of subsystems which include the descending pain modulatory system comprised of the PAG and rostral ventromedial medulla (RVM), as well as the reward-motivation network, including the PFC, NAc, and the ventral tegmental area (VTA). Not surprisingly, this interconnected group of brain regions also forms the basis for understanding the pathophysiology of depression [25].

Preclinical studies focusing on the affective consequences of neuropathic pain have begun to link the aforementioned circuit changes with behavior outcomes in pain states. These studies have provided insights into the potential function of regions involved in the anxio-depressive components of chronic pain—most notably, the ACC, IC, hippocampus, amygdala, the NAc, and the VTA (Figure 1) [62]. The ACC, which processes cognitive, emotional, and autonomic functions and interacts with the thalamus and amygdala, has been implicated in both pain processing and depression [63, 64]. Hypermetabolism and diminished volume of the ACC have both been observed in depressed patients and ACC changes, including altered membrane potential oscillations, have separately been observed in the CCI neuropathic pain model [65]. Recruitment of the ACC may play multiple roles in the experience of pain and pain-induced depression, from anticipation to aversion. In the SNI neuropathic pain model, for example, the volume of the ACC was found to diminish only after a delay, at the same time as the onset of anxio-depressive symptoms, suggesting that structural changes in the ACC potentially represent certain depressive changes in response to pain [66]. The ACC response to pain, moreover, has been shown to be potentiated by amputation [67]. Lesion studies have moreover confirmed that the ACC is necessary for the aversive component of neuropathic pain [68]. Additionally, the ACC has been implicated in pain expectation in imaging studies [69]. The IC, meanwhile, has been strongly implicated in processing pain intensity and interacts with several other key pain regions, including the PFC, ACC, S2, and amygdala (Figure 1). Because of the complexity of the IC's connections with other relevant regions, its role in processing affective or depressive symptoms of pain remains a strong possibility. In addition, IC activity has been tied to both antinociceptive and pronociceptive functions [70].

The hippocampus and amygdala are two additional structures that likely play a role in pain-induced depressive behaviors. The hippocampus, a region known for its role in learning and memory, has been well-studied in the regulation of depressive phenotype. Recent animal studies have shown that cognitive and affective processes are impaired in chronic neuropathic pain conditions and that this impairment corresponds with a decreased hippocampal volume [71]. Neurometabolic changes that can result in decreased serotonin levels in the hippocampus have also been demonstrated to cause depressive symptoms in an inflammatory pain model in rodents [52]. Moreover, studies have demonstrated that pain-induced changes in the hippocampus correlate with anxiety-like behaviors commonly associated with the depressed state [71]. In contrast to decreased hippocampal volume, neuropathic pain has been observed to increase the volume and neuronal proliferation in both the central and basolateral nuclei of amygdala [72]. Given the well documented role of the amygdala in fear and in mood disorders, the amygdala has been suggested as a strong candidate for mediating depression-like symptoms resulting from pain [73–75]. Indeed, a number of studies have demonstrated that the amygdala is crucial in maintaining anxio-depressive and nocifensive behaviors in persistent inflammatory and neuropathic pain states [76–80].

Recent studies of the mesolimbic pathway, including the PFC, VTA, and NAc, have indicated a prominent role for these regions in pain processing, particularly in the depression-like symptoms resulting from chronic pain. The PFC, VTA, and NAc play crucial roles in reward processing, and these regions are also activated by nociception [81–83]. The PFC-VTA-NAc axis is critical for the maintenance of normal hedonic experience and motivation, and hence this reward circuitry also plays a part in the pathogenesis of depression [56]. A recent study showed that chronic pain induces the synaptic incorporation of calcium permeable *α*-amino-3-hydroxy-5-methyl-4-isoxazolepropionic acid (AMPA) receptors in the NAc and that transmission through these receptors has important antidepressant properties in the chronic pain state [58]. Meanwhile, the PFC has been found to play a role in pain through its connection with the NAc (Figure 1) [23]. It has been shown recently that basal dendrites in the PFC of SNI-treated rodents are longer and have more branches than those of control animals, and spine density is also selectively increased in basal dendrites of these neurons from SNI rats [84]. Thus, changes in the circuit that links the PFC, VTA, and NAc may play an important role in the relationship between pain and depression.

## 5. Molecular Mechanisms That Underlie the Long-Term Plasticity of Pain-Induced Depression

### 5.1. Glutamate Signaling

The current glutamate hypothesis for depression posits that long lasting alterations in glutamate signaling contribute to the regulation of depressive phenotype [85]. AMPA receptors are the primary mediators of excitatory synaptic transmission, and they are composed of four subunits, GluA1–4. Reduced GluA1 levels in the amygdala, PFC, and hippocampus have been found in several rodent stress models of depression [86–88]. In the NAc, meanwhile, lower levels of GluA2-containing AMPA receptors have been described in depressive states [89, 90]. Several lines of evidence suggest the importance of AMPA receptor signaling in depression. First, GluA1 knockout mice display vulnerability to depression [91]. Second, antidepressants can increase GluA1 and GluA2 expression in the PFC and NAc [92, 93]. Lastly, AMPA potentiators which directly increase AMPA receptor activities have been shown to have antidepressant properties [50].

Glutamate signaling has been studied in animal pain models as well. In the classic PAG-RVM-spinal descending pathway [94–96], neurons from the PAG form glutamatergic projections through AMPA receptors on GABAergic cells in the RVM to inhibit dorsal horn neurons [97]. Not surprisingly, the administration of glutamate into the PAG is known to produce analgesia [98–100]. In the RVM, meanwhile, AMPA receptor upregulation mediates analgesia in inflammatory pain states [101, 102], whereas their downregulation in neuropathic pain causes hyperalgesia [103]. Thus, transmission through AMPA receptors is required for the intact PAG-RVM descending pathway [104, 105]. A second pain modulating center that depends on glutamate signaling is the NAc. The NAc provides pain-induced analgesia, in part through its projection to the RVM [106]. Intra-NAc administration of AMPA receptor antagonists, however, can disrupt this pain-induced analgesic mechanism [107]. Furthermore, chronic pain has been shown to decrease vesicular glutamate transporter (VGLUT) 1 and 3 levels in the NAc, suggesting a decrease in glutamate signaling in this region [108]. The most direct evidence for the analgesic effects of glutamate signaling comes from a study that showed AMPAkines, positive allosteric modulators for AMPA receptors, have antinociceptive properties in persistent inflammatory and neuropathic pain conditions [53].

At the same time, however, chronic inflammatory pain increases trafficking of GluA1 AMPA receptor subunits but decreases GluA2 delivery [109–111], leading to the formation of GluA2-lacking receptors [112]. Similarly, AMPA receptor signaling in the ACC and amygdala has also been suggested to increase synaptic plasticity and confer hyperalgesia [113–117]. Thus, in chronic pain conditions, AMPA receptor signaling plays both pronociceptive and antinociceptive roles, depending on the target CNS regions.

An emerging number of studies have demonstrated that glutamate signaling also plays an important role in specifically mediating the depressive symptoms of pain. For example, a study on AMPA receptor subunit trafficking showed that chronic neuropathic pain selectively increases GluA1 levels at the synapse of the NAc, leading to the formation of GluA2-lacking, or calcium permeable AMPA receptors (CPARs) [58]. Glutamate transmission through these newly formed CPARs, in turn, relieves the depressive symptoms of pain without altering pain sensitivities. In addition, pharmacologic studies using ketamine and AMPAkines, two classes of drugs that increase glutamate signaling through AMPA receptors [92, 118, 119], have demonstrated that drugs that increase glutamate signaling in the brain can also treat the depressive symptoms of chronic pain [49, 53]. Thus, glutamate signaling, particularly signaling through AMPA receptors, plays a key role in regulating pain, depression, and depression in the context of chronic pain.

### 5.2. Modulatory Neuropeptides (Serotonin, Dopamine, and Norepinephrine)

Modulatory neuropeptides have long been studied in the context of both depression and pain. Norepinephrine (NE) neurons are found in the locus ceruleus (LC), and decreased NE signaling is known to be associated with depression [120–124]. The LC releases NE into multiple regions in the brain, including the cerebral cortex, limbic system, and spinal cord. Originally thought to regulate attention and fight-or-flight responses, the NE system has been shown to affect a wide range of cognitive and affective functions. Activation of the descending modulatory pathway from the RVM and PAG, meanwhile, can release NE into the spinal dorsal horn. The binding of NE to the spinal alpha2 receptors has been found to exert antinociceptive effects [125]. Thus, noradrenergic system can have a profound influence on the pathogenesis of pain and depression.

Dopamine (DA) neurons are located in the VTA. Dysfunctional DA signaling can cause depression [126–130]. Dopamine dysfunction has also been associated with increased pain sensitivity in several chronic pain conditions, including headache, fibromyalgia, and osteoarthritis [131–134]. Interestingly, Parkinson's disease, which presents a classic hypodopaminergic state, is associated with increased incidence of both depression and chronic pain [135, 136]. While the molecular mechanism for the role of DA signaling in pain-induced depression remains incompletely characterized, it is thought that dopaminergic signaling in the PFC and NAc is critical for the maintenance of normal hedonic drive, as well as working memory, concentration, and locomotion. Thus, dysfunction of the DA system results in symptoms of anhedonia, reduced concentration, sleep disturbance, and psychomotor retardation, all salient symptoms of pain-induced depression [127, 137].

Serotonin (5-HT) cells are located in the dorsal and medial raphe nuclei. The role of 5-HT in depression is well-described [120, 121, 138, 139]. 5-HT is projected widely throughout the brain to modulate synapses as well as the release and function of NE and DA, and its activity in the PFC, ACC, VTA, and NAc is thought to influence mood and affect. In addition, 5-HT has also been shown to be critical for descending modulation of pain [140–144]. Activation of the descending projections from the RVM, which includes nucleus raphe magnus, produces 5-HT release in the spinal dorsal horns [97, 145–148]. The RVM provides both descending inhibition and facilitation of pain, through the activation of “on” and “off” cells, respectively [148–154]. Thus, 5-HT signaling can exert both antidepressant and antinociceptive properties by modulating synaptic connectivity and the other monoamine signaling in various regions in the brain. A recent study examined the role of brain indoleamine 2,3-dioxygenase (IDO1) in the comorbidity of pain and depression [52]. IDO1 is a rate-limiting enzyme in the metabolism of tryptophan, a precursor to serotonin, and IDO1 activity has been linked to decreased 5-HT content. This study found that inhibition of hippocampal IDO1 activity attenuated pain-induced depression, thus providing further evidence that 5-HT signaling can exert control over the comorbidity of pain and depression.

### 5.3. Neurotrophic Factors and Neuromodulatory Lipids

Brain-derived neurotrophic factor (BDNF) promotes the formation of synaptic plasticity [155]. BDNF binds to two receptors on the surface of cells: TrkB and the low-affinity nerve growth factor receptor. TrkB is a receptor tyrosine kinase, and the binding of BDNF to TrkB promotes the formation of long-term potentiation at synapses as well as de novo neurogenesis. Decreased BDNF levels have been identified in patients who have suffered from major depressive disorder, whereas patients who underwent successful antidepressant treatment have shown increased BDNF levels [156–158]. Elevated BDNF levels have been associated with fibromyalgia, whereas a decreased level has been found in patients with chronic migraine [159, 160]. BDNF val66met polymorphism, in particular, has been identified in patients with chronic abdominal and pelvic pain [161, 162]. BDNF has been shown to regulate both peripheral and spinal sensitivity to chronic pain in various animal models [163]. Furthermore, in the hippocampus, decreased BDNF has been found in chronic inflammatory pain states, and this decrease has been shown to be responsible for depression-like behaviors [164]. Thus, by modulating synaptic plasticity throughout the peripheral and central pain circuits, BDNF can alter pain sensitivity and, more importantly, the level of pain-induced depression.

The endocannabinoid system is a signaling system comprising the G-protein coupled cannabinoid CB1 and CB2 receptors, their intrinsic lipid ligands, endocannabinoids such as the N-arachidonoyl ethanolamide (anandamide) and the 2-arachidonoyl glycerol, and associated proteins (transporters, biosynthetic, and degradative enzymes) [165]. The CB1 receptor couples to both G_i/o_ proteins which function to inhibit adenylyl cyclase activity, activate potassium channels, and inhibit voltage-gated calcium channels, while the CB2 receptor is known to couple to G_i_ proteins. The CB1 receptor is located predominantly on presynaptic axon terminals, and it regulates calcium influx and subsequent neurotransmitter release to influence synaptic plasticity. CB1 knockout mice become anhedonic during chronic mild stress, and they also demonstrate decreased motivation, indicative of the depressive phenotype [166, 167]. These changes are likely caused by alterations in CB1 signaling that help to maintain normal synaptic efficiency in the PFC, amygdala, hippocampus, and NAc [168–171]. Both CB1 and CB2 receptors are abundantly expressed in motor and limbic regions and in areas that are involved in pain transmission and modulation, including the PAG, RVM, spinal cord dorsal horn, and the peripheral nerve. In the RVM, CB1 signaling can alter the “on” and “off” cell balance to favor descending inhibition. In the spinal dorsal horn, it can decrease* N*-methyl-D-aspartic acid or* N*-methyl-D-aspartate (NMDA) receptor activation and decrease noxious stimulus-evoked firing of wide dynamic range neurons [172]. Endocannabinoid signaling can also inhibit neuropeptide release from primary afferent fibers in response to acute noxious stimulus. Thus, endocannabinoid signaling plays important roles in regulating both depression and pain. Interestingly, a recent study showed that a CB2-selective agonist GW405833 specifically relieves depressive symptoms of chronic neuropathic pain in rats, but it has no antidepressant effects in rats that do not experience pain [173]. This finding not only indicates that pain can induce depression, but it provides evidence that pain-induced depression may have unique physiologic and molecular mechanisms.

### 5.4. Transcriptional, Translational, and Epigenetic Mechanisms

Transcriptional mechanisms have been well studied in animal models of depression, and genome wide arrays have begun to uncover genetic bases for depression in patients who suffer from major depressive disorders. The best characterized transcriptional mechanism controlling depression-like behaviors involves CREB. The role of CREB on depressive symptoms varies depending on the specific brain regions. Increased transcriptional activity in the hippocampus induced by CREB has been demonstrated to have antidepressant effects. In contrast, CREB signaling in the NAc promotes stress susceptibility and induces anhedonic behaviors [174]. Prominent targets for CREB include potassium channels, glutamate receptors, dynorphin, and other neuropeptides [175–177]. By acting on these receptors and peptides, CREB can increase the intrinsic excitability of cortical, hippocampal, and accumbal neurons and promote glutamatergic plasticity, which will in turn serve as circuit level regulators for affect and behavior. The role of CREB is well documented in animal pain models as well. A number of studies have shown that CREB signaling in the hippocampus, cortex, and NAc can alter pain sensitivity [178–180]. Furthermore, a recent study demonstrated that CREB signaling in the ACC promotes the negative affective experience of pain, as manifested by conditioned place avoidance [181]. Thus CREB is likely an important mediator of depressive symptoms of pain.

Translational regulators, especially mTOR, have been actively investigated in both pain and depression models in rodents. mTOR signaling in the PFC has been shown to give rise to dendritic growth and upregulation of the synaptic machinery, which underlie the antidepressant effect of ketamine [92, 182]. Recently, mTOR signaling in the hippocampus has also been found to regulate pain-related synaptic plasticity [183]. Thus, translational regulation provides another level of control for the comorbidity of pain and depression.

In addition to transcriptional and translational control of synapse and neuronal excitability, proteins that posttranslationally modify histone, DNA, and recruit large sets of coactivators or corepressors of genes have come under active scrutiny over the last two decades. Such epigenetic mechanisms have been identified to play significant roles in the pathogenesis and regulation of depression and pain. For example, local inhibition of certain histone deacetylases (HDACs) or DNA methyltransferases (DNMTs) and conversely local activation of histone methyltransferases (HMTs) in the NAc have been found to have antidepressant effects in various animal models of depression [184–186]. Similarly, alterations of HDACs and DNMTs in the cortex and hippocampus have also been implicated in depression-like behaviors [187–189]. Epigenetic mechanisms have been demonstrated to alter pain sensitivity at both spinal and peripheral levels [190]. In addition, studies have begun to reveal that changes in HDACs and DNMTs in the hypothalamus, cortex, and brain stem can regulate both sensory and affective pain behaviors [191–193]. Particularly, altered DNA methylation of the corticotropin releasing-factor genes in the hypothalamic-pituitary-adrenal axis has been shown to result in stress-induced pain hypersensitivity, raising the possibility that central epigenetic mechanisms can play a role in the relationship between pain and depression [191].

These molecular mechanisms are summarized in Table 1.

## 6. Treatment for the Comorbidity of Pain and Depression

A line of clinical evidence that supports the close pathological relationship between chronic pain and depression comes from the drugs that can treat both conditions. Currently, two classes of antidepressants, serotonin, and norepinephrine reuptake inhibitors (SNRIs) and tricyclic antidepressants (TCAs) are Federal Drug Administration (FDA) approved for the treatment of depression as well as a variety of chronic pain conditions. Furthermore, experimental treatments, including ketamine and deep brain stimulation, have been demonstrated to have efficacy in treating both pain and depression.

### 6.1. Serotonin and Norepinephrine Reuptake Inhibitors (SNRIs)

Serotonin reuptake inhibitors (SSRIs) and serotonin and norepinephrine reuptake inhibitors (SNRIs) are mainstay treatment options for depression. By inhibiting the reuptake of serotonin, SSRIs can increase the extracellular levels of these neurotransmitters [194]. SNRIs, in addition, increase the level of norepinephrine as well. SSRIs are currently the first-line treatment for depression. While the exact targets for these drugs are not known, it is thought that they exert antidepressant effects by augmenting synaptic plasticity in the cortex, hippocampus, striatum, and a number of other brain regions. In addition, SSRIs can promote neurogenesis in the hippocampus [195]. However, the efficacy of SSRIs in pain has been disappointing, with number needed to treat greater than 15 for most of the drugs in this class. SNRIs, in contrast, have been highly successful in treating a wide range of pain, particularly neuropathic pain, including diabetic neuropathy, postherpetic neuralgia, and fibromyalgia [196–200]. Thus, likely SNRIs modulate the NE signaling pathway in the CNS to decrease pain and pain-induced depression. One possible mechanism is the activation of the descending modulatory pathway from the RVM and PAG to the dorsal horn neurons [125]. On the other hand, there is increasing evidence that the pathogenesis of fibromyalgia strongly involves abnormal processing in the brain, and the incidence for the comorbidity of fibromyalgia and depression is particularly high [201, 202]. Thus, SNRIs likely can also alter NE signaling and synaptic transmission in the brain to decrease pain, depression, and other associated affective symptoms in fibromyalgia patients [202]. Currently, duloxetine is the first-line treatment option for fibromyalgia. For neuropathic pain in general, the number needed to treat is approximately 2–5 for SNRIs [203]. It is interesting to note, however, that the dose of SNRIs for chronic pain treatment is approximately half of what is used for the treatment of major depressive disorder.

### 6.2. Tricyclic Antidepressants (TCAs)

Tricyclic antidepressants (TCAs) are an older class of antidepressants. These drugs block the serotonin transporter and norepinephrine transporter to increase the synaptic concentration of these neurotransmitters [194]. Thus, the pharmacologic mechanisms for SNRIs and TCAs are similar. TCAs have been largely replaced by the SSRIs and SNRIs as the first-line treatment for depression due to their more serious side effect profile and poorer tolerability at higher doses. Over the past two decades, however, TCAs have been widely used in the treatment of a number of chronic pain conditions, most notably neuropathic pain conditions [199]. They are used for essentially the same pain syndromes as SNRIs, including diabetic neuropathy, postherpetic neuralgia, and fibromyalgia as well as chronic low back pain with an element of radiculopathy. The number needed to treat pain for the TCAs is comparable to that of the SNRIs [204]. Similar to the SNRIs, the dose of TCAs for chronic pain treatment is less than half of what is used for depression. The commonly used TCAs include amitriptyline, nortriptyline, and desipramine.

### 6.3. Ketamine

Ketamine is classified as a dissociative anesthetic, and it is FDA approved as an anesthetic as well as an analgesic agent. Ketamine affects the function of a number of receptors and channels. It is known to block NMDA receptors. It is also an agonist for dopamine D2 receptors and blocks dopamine reuptake. More recently, ketamine has been shown to increase presynaptic glutamate release and to activate mTOR to promote the translation of GluA1 AMPA receptor units at the postsynaptic site [92]. Recently, ketamine has emerged as a fast onset, long-lasting, and potent antidepressant. A single subanesthetic dose of IV ketamine has rapidly and reproducibly decreased depressive symptoms in treatment-resistant depressed patients, with antidepressant responses detected within 1-2 hours after infusion, maintained in a majority of patients for at least 24 hours, and in some cases for up to 7 days [205–207]. While the mechanism of antidepressant action for ketamine remains incompletely understood, it likely involves an increase in AMPA receptor signaling, an increase in BDNF levels, and an overall increase in synaptic plasticity in the cortex and hippocampus [92, 208, 209]. In animal studies, a subanesthetic dose of ketamine has been shown to relieve pain-induced depression for up to five days after a single administration [49], and this time frame is comparable to studies of stress-induced depression models as well as human studies of depression. Ketamine has long been used as an analgesic for acute pain. Recently, sub-anesthetic-dose ketamine infusion has been used for the treatment of refractory neuropathic pain syndromes with significant depressive comorbidities, most notably the complex regional pain syndrome [210–213]. In these patients, repeated infusions of subanesthetic doses of ketamine have been shown to effectively treat pain as well as associated affective comorbidities, including depressed mood. In a number of these studies, the effect has lasted weeks to months.

### 6.4. Future Therapeutic Developments

Despite of these drugs discussed above, pharmacologic options for the comorbidity of pain and depression remain fairly limited. Based on our current understanding of circuit and molecular mechanisms for the comorbidity of pain and depression, however, two future therapeutic approaches hold promise. The first approach is to use neuromodulation (such as deep brain stimulation (DBS) or transcranial magnetic stimulation or even biofeedback or cognitive behavioral therapy) to target the converging circuit pathways in the brain that are responsible for pain as well as depression. Our knowledge of the converging pain and depression circuitry suggests that certain brain regions, including the dorsolateral PFC, hippocampus, and NAc, can be potential targets for neuromodulation. DBS targeting some of these regions, in particular the PFC, can significantly alter long-term neural plasticity and has been under intense and active investigation for refractory depression [214–216]. However, neuromodulation treatment for pain has been limited to spinal cord and peripheral afferent stimulations [217]. In the future, DBS targeting the PFC and NAc should be considered in preclinical and clinical studies for the comorbidity of pain and depression. A second hypothetical therapeutic approach that holds promise is targeting the excitatory neurotransmission that can treat both pain and depression. In this regard, AMPA receptors are ideal targets. A recent study shows that AMPAkines, drugs that potentiate AMPA receptor function, can relieve depressive symptoms of pain [50]. Furthermore, ketamine, which increases AMPA receptor signaling, has also been shown to relieve pain-induced depression in animal models [49]. Thus, future clinical studies are needed to further evaluate the efficacy of drugs such as AMPAkines and ketamine in the treatment of comorbidity of pain and depression. Finally, an intriguing class of drugs are HDAC inhibitors which, by modifying genomic expression at a large scale, can profoundly affect both the circuit and molecular basis of pain and depression. HDAC inhibitors have been shown to relieve pain in neuropathic and inflammatory models of pain [151, 218]. They have antidepressant effects in animal models of depression as well [186]. HDAC inhibitors have already been approved for the treatment of some cancers, and the HDAC inhibitor valproic acid is used as a mood stabilizer [154]. Future studies are needed to determine whether inhibition of a single or multiple HDAC isoforms is most efficacious for the treatment of pain and depression.

## 7. Conclusion

Pain and depression are important comorbidities. Both clinical and preclinical studies clearly indicate that pain can cause depression, and that depression can worsen pain behaviors. The CNS undergoes long-term plastic changes associated with chronic pain and depression. Circuit and molecular mechanisms that underlie this plasticity have begun to emerge. Some of the successful current therapeutic approaches have validated these mechanisms, and future therapies based on such mechanistic understanding can be developed to better serve clinical needs.

## Figures and Tables

**Table 1:** Molecular mechanisms in pain and depression.

Molecular marker	Depression	Pain
Glutamate	(i) Reduced GluA1-containing AMPA receptors in amygdala, PFC, and hippocampus (ii) Reduced GluA2-containing AMPA receptors in NAc (iii) Ketamine's antidepressant actions likely include increases in AMPA receptor signaling	(i) AMPA receptor upregulation in RVM mediates analgesia; AMPA receptor downregulation in RVM causes hyperalgesia (ii) Reduced VGLUT1 and 3 levels in NAc (iii) Reduced GluA2-containing AMPA receptors in NAc (iv) Ketamine's analgesic actions likely due to NMDA antagonism

Norepinephrine	Decreased signaling in LC	Activation of RVM and PAG causes norepinephrine release and antinociception

Dopamine	Decreased signaling in VTA and NAc	Decreased signaling in the NAc

Serotonin	Altered signaling in PFC, ACC, VTA, and NAc	Can both inhibit and facilitate pain by projection to off and on cells in the RVM

BDNF	Decreased serum levels	(i) Elevated serum levels in fibromyalgia (ii) Decreased serum levels in migraine

Endocannabinoids	CB1 knockout mice display depressive phenotype	(i) CB1 signaling in the RVM favors descending inhibition (ii) CB1 signaling in the spinal dorsal horn decreases NMDA receptor activation

CREB	(i) Increased activity in hippocampus has antidepressant effects (ii) Signaling in NAc induces anhedonic behaviors	Signaling in the hippocampus, cortex, and NAc can alter pain sensitivity

mTOR	Signaling in PFC underlies antidepressant effect of ketamine	Signaling in the hippocampus regulated pain-related synaptic plasticity

Epigenetic	(i) Inhibition of HDACs and DNMTs in NAc has antidepressant effects (ii) Activation of HMTs in NAc has antidepressant effects (iii) Alterations of HDACs and DNMTs in cortex and hippocampus implicated in depressive behaviors	Changes in HDACs and DNMTs in hypothalamus, cortex, and brain stem can regulate sensory and affective pain behaviors

AMPA: *α*-amino-3-hydroxy-5-methyl-4-isoxazolepropionic acid; BDNF: brain-derived neurotrophic factor; CB: cannabinoid receptor; CREB: cAMP response element-binding protein; DNMT: DNA methyltransferase; HDAC: histone deacetylase; HMT: histone methyltransferase; LC: locus ceruleus; mTOR: mammalian target of rapamycin; NAc, nucleus accumbens; NMDA: N-methyl-D-aspartate; PAG, periaqueductal gray; PFC: prefrontal cortex; RVM: rostral ventromedial medulla; VGLUT: vesicular glutamate transporter; VTA: ventral tegmental area.
